# Seroprevalence of SARS-CoV-2 IgG and associated factors among people living with HIV over the first 12 months following the outbreak of COVID-19 in Burkina Faso, a sub-Saharan African country

**DOI:** 10.1371/journal.pone.0286665

**Published:** 2023-06-14

**Authors:** Odilon D. Kaboré, Armel Poda, Cheick Ahmed Ouattara, Fernand N. Michodigni, Abdoul-Aziz Belem, Yacouba Sawadogo, Jacques Zoungrana, Lokman Galal, Clément Z. Meda, Sylvain Godreuil, Abdoul-Salam Ouédraogo

**Affiliations:** 1 Department of Bacteriology and Virology, Souro Sanou University Hospital, Bobo Dioulasso, Burkina Faso; 2 Laboratory of Emerging and Re-emerging Pathogens, School of Health Sciences Nazi Boni University, Bobo Dioulasso, Burkina Faso; 3 Superior Institute of Health Sciences, Centre Hospitalier Universitaire Souro Sanou, NAZI BONI University, Bobo-Dioulasso, Burkina Faso; 4 Department of Infectious Diseases and Tropical Medicine, Souro Sanou University Hospital, Bobo Dioulasso, Burkina Faso; 5 Muraz Center, Bobo Dioulasso, Burkina Faso; 6 Laboratoire de Bactériologie, Centre Hospitalier Universitaire de Montpellier, Montpellier, France; 7 Maladies Infectieuses et Vecteurs: Ecologie, Génétique, Evolution et Contrôle (MIVEGEC), Université de Montpellier—Institut de Recherche pour le Développement (IRD)—Centre National de la Recherche Scientifique (CNRS), Montpellier, France; University of Ilorin, NIGERIA

## Abstract

**Objective:**

This study aimed to evaluate the seroprevalence of anti-SARS-CoV-2 IgG and factors associated with the infection among PLWHIV over the first 12 months following the outbreak of COVID-19 in Burkina Faso.

**Design:**

A retrospective cross-sectional study of plasma samples collected from March 9, 2020, and March 8, 2021, at the outpatient HIV referral center, before the introduction of the SARS-CoV-2 vaccine in Burkina Faso.

**Methods:**

Anti-SARS-CoV-2 IgG were detected in plasma using DS-ЕIA-ANTI-SARS-CoV-2-G (S) kit. Logistic regressions were used to compare SARS-CoV-2 specific immune responses between groups and within subgroups.

**Results and discussion:**

A total of 419 plasma were subjected to serological diagnosis. None of the participants was vaccinated against COVID-19 during the period of sample collection, and 130 samples were positive for anti-SARS-CoV-2 IgG, giving a prevalence of 31.0% (95% CI 26.6–35.7). The median CD4 cell count was 661 cells/μL (IQR,422–928). Retailers had half the risk of being infected compared to housemaids with an OR of 0.49 (*p* = 0.028, 95% CI 0.26–0.91). Likewise, the risk of infection was 1.69 times higher in patients on integrase inhibitors compared to that of patients on non-nucleoside reverse transcriptase inhibitors (*p* = 0.020, 95% CI 1.09–2.63).

**Conclusion:**

Our study reveals a high seroprevalence among PLWHIV to SARS-CoV-2 during the first year of the pandemic. In addition, PLWHIV on integrase inhibitors are 1.69 times more likely to be infected than PLWHIV on non-nucleoside inhibitors, and this observation remains an intriguing topic that still needs to be clarified.

## Introduction

On December 31, 2019, cases of pneumonia of unknown etiology and severe acute respiratory infection appeared in Wuhan, China and the causative agent was identified later as the Severe Acute Respiratory Syndrome Coronavirus 2 (SARS-CoV-2) [1]. The virus quickly spread worldwide and become one of the greatest public health emergencies [2, 3].

Previous studies reported that people with underlying medical conditions such as diabetes, hypertension, and cardiovascular disease are likely to be infected with SARS-CoV-2 and are at higher risk of death from complications associated with COVID-19 [4, 5]. Accordingly, People Living with Human Immunodeficiency Virus (PLWHIV) are a particular subset of vulnerable populations who often develop chronic diseases and co-morbidities such as tuberculosis [6]. This group of people may also be exposed to poor outcomes from a COVID-19 infection. Surprisingly, there are divergent opinions about the potential worsening symptoms and conditions in PLWHIV co-infected with SARS-CoV-2 compared to the general population [7–10]. Indeed, some studies reported that the susceptibility of PLWHIV to SARS-CoV-2 infection is similar to the general population whereas others indicated that HIV-infected individuals were at higher risk of coming down with COVID-19 when compared to HIV-negative individuals [11, 12]. It was, therefore, hypothesized that an impaired immune system could hamper the cytokine storm that is associated with severe symptoms of COVID-19, which may logically favour PLWHIV with a low CD4+ cell count [13].

Modelling studies suggest that the COVID-19 pandemic could cause an excess of 0.5 million HIV-related deaths by 2025 due to the collapse of health systems or the disruption of prevention and treatment services [9, 14]. Accordingly, Sub-Saharan Africa is one of the focal areas of the HIV epidemic and accounts for more than 70% (25.4 million) of PLWHIV with Burkina Faso having 0.7% of that number. The country has been facing an unprecedented healthcare crisis since the outbreak of COVID-19 and estimating COVID-19 cumulative incidence remains problematic due to challenges in contact tracing, routine surveillance systems and laboratory testing capacities and strategies [15]. As a result, clinicians and researchers working on SARS-CoV-2 infection find it difficult to understand the burden of SARSCoV-2 in PLWHIV. Unfortunately, the confirmation of COVID-19 cases using a molecular diagnostic test (PCR) is under-utilized due to lack of screening among mildly affected or asymptomatic individuals [16]. In addition, RT-PCR testing could yield false-negative results, mainly due to preanalytical problems [17, 18].

Thus, to allow a correct estimation of the spread of the disease within a population, cross-sectional serological studies (*e*.*g*. SARS-CoV-2 IgG ELISA) are required to grasp the overall picture of SARS-CoV-2, monitor changes in seroprevalence over time, anticipate the dynamics of SARS-CoV-2 propagation and plan an adequate public health response to the ongoing pandemic as recommended by WHO [19]. Except for few SARS-CoV-2 seroprevalence studies that have been carried out with samples collected in 2020 among PLWHIV in Munich (Germany) [20], Spains [21], and Ouagadougou, Burkina Faso [22] with an estimated seroprevalence of 1.5%, 8.5% and 18% respectively, seroepidemiological data on SARS-CoV-2 infection in PLWHIV remain scarce to our knowledge in Sub-Saharan African country before the introduction of the SARS-CoV-2 vaccine.

Hence, the current study aimed to evaluate the seroprevalence of anti-IgG against SARS-CoV-2 Spike positivity and associated factors in PLWHIV over the first 12 months following the outbreak of COVID-19 in Burkina Faso, a sub-Saharan African country.

## Methods

### Study area/site

The present study was a sub-study of the ‘‘Faso covid” protocol that has been published [23]. Briefly, the study was carried out at the Souro Sanou University Hospital Center, a reference center for the care of PLWHIV in the Hauts-Bassins region and responsible for the follow-up of at least 10.000 PLWHIV aged 18 years and older. The hospital is located in Bobo Dioulasso, the economic capital of Burkina Faso. It is mainly a referral health centre for four of the thirteen regions of Burkina Faso with a population of about six million.

### Study design and participant selection

We conducted a retrospective cross-sectional serological study of residual PLWHIV plasma from specimens collected for routine plasma HIV-RNA viral load measurements between March 9, 2020, and March 8, 2021. This period corresponds to the date of detection of the first two cases of COVID-19 (March 9, 2020) and the time before the introduction of the SARS-CoV-2 vaccine in Burkina Faso [24, 25].

The inclusion criteria were *i)* PLWHIV aged 18 years or older followed-up at the outpatient’s HIV referral center of Souro Sanou University Hospital Center between March 9, 2020 to March 8, 2021, *ii)* naive to SARS-CoV-2 vaccine, *iii)* having given their informed consent for the use of their medical records and plasma sample for research purposes. In case of several available samples, a random selection was made to obtain samples between the last sample of the year 2020 and the first sample of the year 2021 [23].

The exclusion criteria included *i)* plasma samples which were not linked to clinical/laboratory data registered in the electronic patient files named Evaluation et Suivi Opérationnel des Programmes Esther (ESOPE), *ii)* patients younger than 18 years and *iii)* those who refused to consent [23].

#### Instrument of data collection

After first identifying and including the plasmas selected from the biobank of PLWHIV to perform the ELISA IgG SARS-CoV-2 test, all demographic and medical records were then extracted from the ESOPE database and exported to Microsoft Excel 2016 [20]. All PLWHIV followed up at the Souro Sanou University Hospital Center are registered in a patient database ESOPE. The database is highly secured with controlled access and anonymity. The ESOPE database collects socio-demographic characteristics (age, sex, place of residence, socio-professional category), HIV-related characteristics (period of HIV infection, anti-retroviral treatment, period of HIV treatment and CD4 count) and comorbidities (arterial hypertension, diabetes, and tuberculosis history) of the PLWHIV.

### Sample size calculation and sampling

The number of plasma samples needed for this study was calculated using the formula for descriptive studies:

Sample size (n) = [DEFF*Np(1-p)]/ [(d2/Z21-α/2*(N-1) +p*(1-p)] where:

N = Population size (10.000)

p = Estimated seroprevalence rate of 50%

d = Precision = 0.05 (5% margin of error)

DEFF = Design effect (1)

Z1-α/2 = critical value for normal distribution at 95% confidence level (1.96)

Thus, an estimated minimum sample size of 370 participants was obtained using Open Epi online software https://www.openepi.com/SampleSize/SSPropor.htm. This number was increased to at least 400 PLHIV to take into account clinical/biological data not available (*e*.*g*., CD4 count) for some patients in the ESOPE database.

We performed a simple random sampling of patients with biological tests during the period from the unique identifier database.

### Laboratory analyses and detection of anti-SARS-CoV-2 IgG

Blood samples were collected by venepuncture in EDTA tubes, which were then processed, and residual plasma samples obtained from plasma HIV-RNA viral load measurements were promptly aliquoted and stored in the biobank at– 80°C until the use. Enzyme-linked Immunosorbent Assays (ELISA)-based quantitative tests using DS-ЕIA-ANTI-SARS-CoV-2-G (S) kit (RPC Diagnostic Systems, Nizhny Novgorod, Russia) was used for the detection of IgG antibody against the SARS-CoV-2 spike protein according to manufacturer’s instructions. According to the manufacturer, samples taken day 7 after symptom onset, DS-ЕIA-ANTI-SARS-CoV-2-G (S) showed a sensitivity of 39.80%, 79.03% for plasma samples taken between day-8 and day-14, and 100% after more than 15 days. The specificity of the test is reported by the manufacturer to be 100% (IC 95% = 99.14–100.00). Results were calculated as: absorbance value of the plasma divided by absorbance value of the calibrators and expressed as extinction ratio. We utilized the manufacturer’s interpretation of the ratio with plasma, <0.8 classified as no antibody present, 0.8 ≤ 1.2 indeterminate, and >1.2 containing antibodies. For the test to be valid, the absorbance value of positive control should not be less than 1.500. For the negative control, the absorbance value should not be more than 0.150. No cross reactivity was found for samples with antibody to microorganisms that could potentially cause false results in the test, including human coronavirus 229 E, OC43, HKU1, NL63, *Plasmodium falciparum*, Respiratory syncytial virus, *Influenza* A and B virus, *Epstein-Barr* virus, HIV, HCV and HBV [26]. We estimated an overall seroprevalence and an additional month-by-month seroprevalence as the number of positive tests/total patients. Seroprevalence was reported as rate and 95% confidence intervals (CI).

### Statistical analysis

Descriptive analysis of individuals’ characteristics was carried out using frequency distributions for categorical variables, while continuous variables (age, time on ART, CD4 cell counts) were summarized as median and interquartile ranges (IQR). We fitted logistic regression models to identify factors that predicted the risk of SARS-CoV-2 infection. Odds ratios (OR) and 95% confident intervals (95% CI) were calculated between the dependent variable (SARS-CoV-2 IgG positive/negative) and each of the independent variables using univariate logistic regression. Independent variables such as sex, arterial hypertension, diabetes and tuberculosis history were binary-coded, whereas anchor drug-based regimen (INSTI-option, NNRTI-option and PI- option), type of HIV (HIV-1, HIV-2, and coinfection HIV-1/2) were considered as three-level explanatory variables. Socio-professional category was six-level explanatory variables. Independent variables with a statistically significant relationship defined as a *p* value < 0.05 were then included in a multiple logistic regression model. Adjusted odd ratios (aOR) and 95% CI were then calculated after adjustment for confounding factors. Statistical analysis was performed in R 4.2.0 software (www.r-project.org).

### Ethics approval and consent to participate

This study was approved by the national committee for health research of Burkina Faso (N° 2021-08-192) in accordance with the Declaration of Helsinki [27], and the principles of Good Laboratory Practice and Good Clinical Practice [28]. The participants issued signed informed consent for the use of their biological samples (plasma aliquots), clinical records and laboratory data in our electronic database ESOPE.

## Results

**Fig 1:**
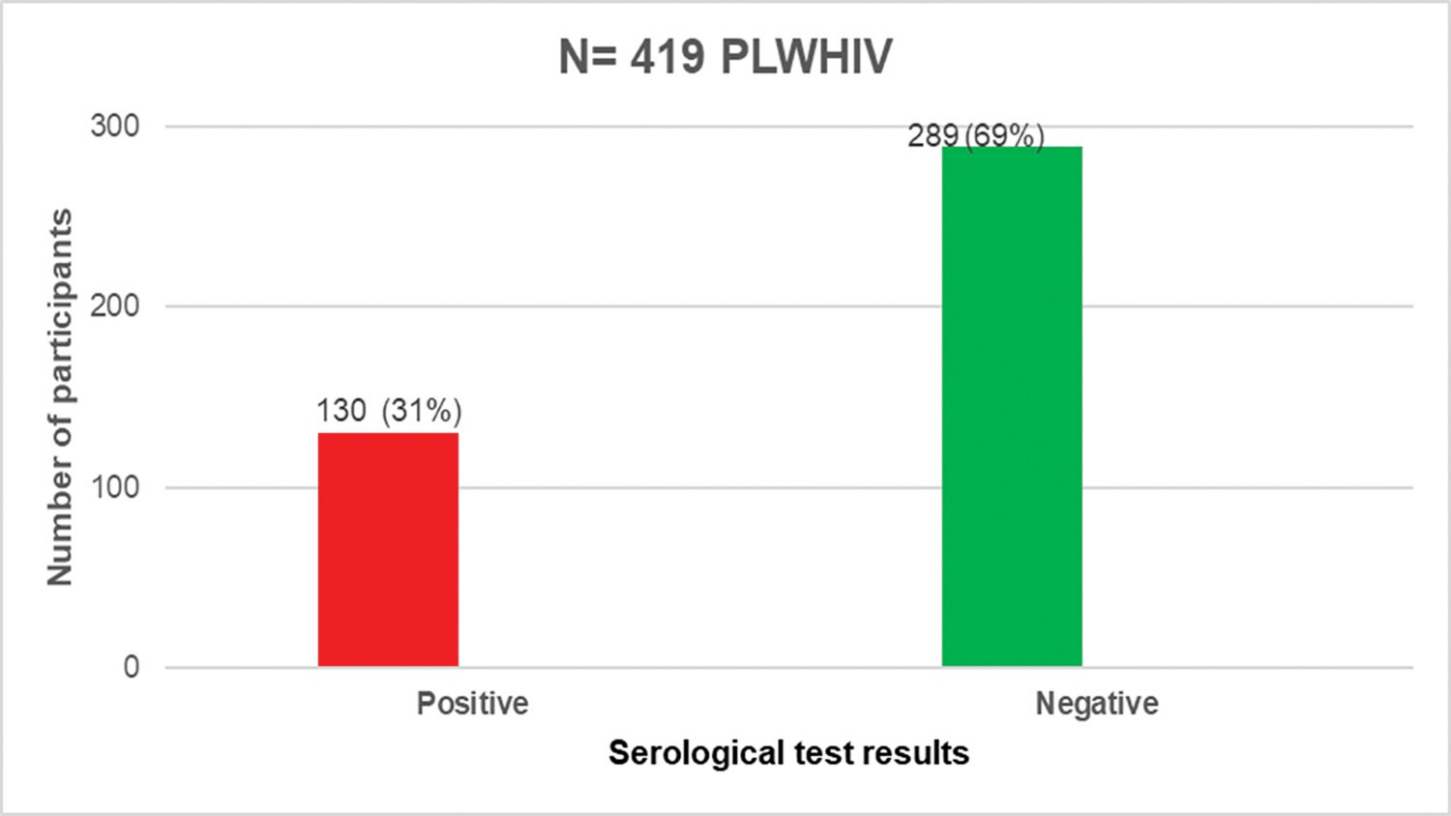
Distribution of Sars-Cov-2 Ig G seropositive and seronegative individuals among the 419 PLWHIV enrolled.

**Fig 2:**
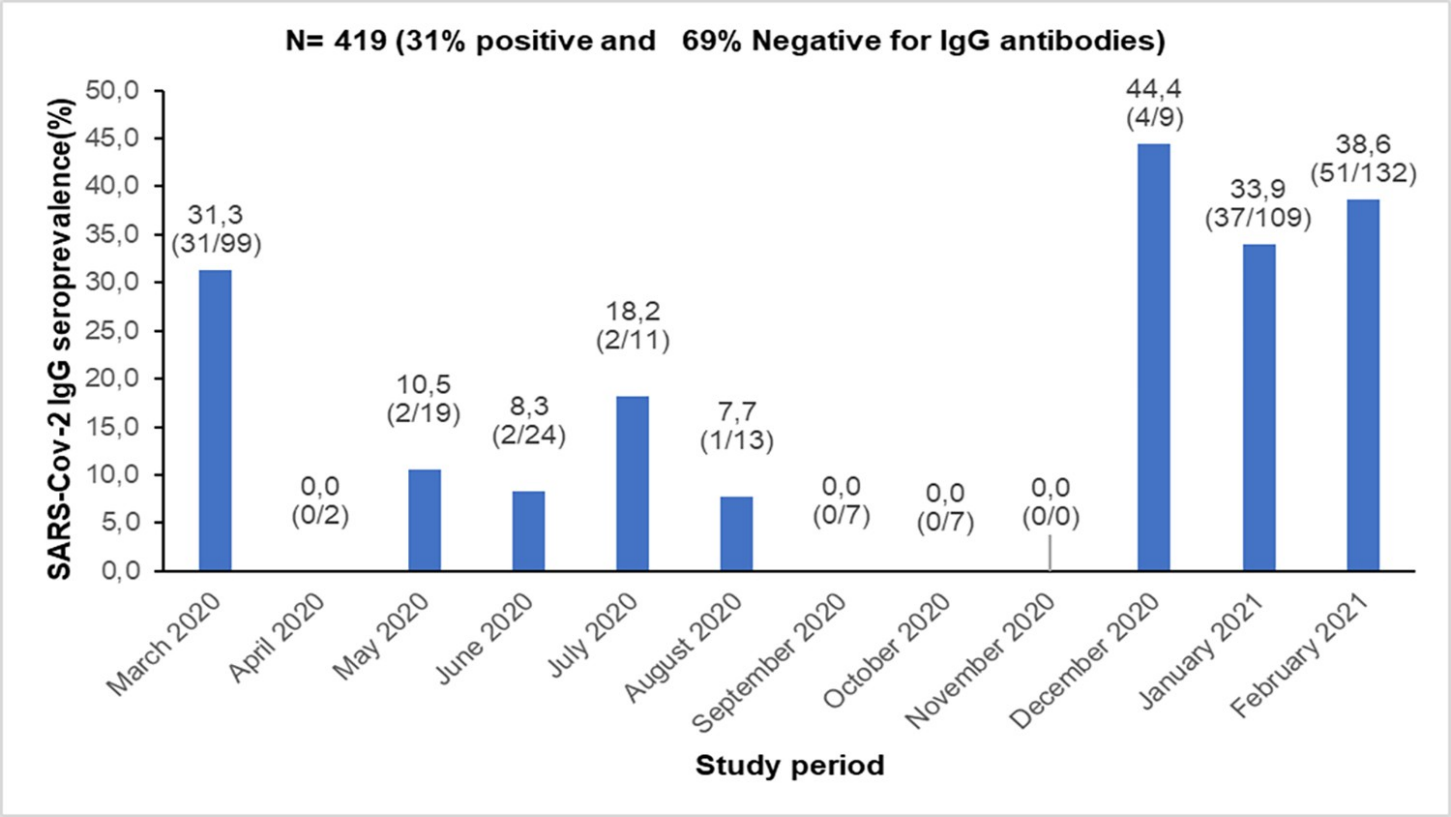
Month by month anti SARS-CoV-2 IgG seroprevalence.

A total of 419 samples from PLWHIV were subjected to the serological diagnostic tests and 26.01% (109/419) were from the male participants. Their median age was 43 years (IQR 35–50), and majority of the participants are from the urban area, representing 90% of the study population. Married among them account for 54.2% and 45.6% had no educational background. In terms of occupations, the study population were housemaids, other workers (bricklayers, plumbers, mechanicians, gardeners and tailors) and retailers representing 47.26%, 19.3% and 19.1% respectively **(Table 1).** About 94% of the study population were infected with HIV-1. The median time from their diagnosis of HIV infection was 11 years (IQR 8–14). All were Antiretroviral Therapy (ART) follow-up patients (median = 11 years, 7–14 IQR) including those on the triple regimen with nucleoside or nucleotide reverse transcriptase inhibitors backbone and an anchor drug (58.9% non-nucleoside reverse transcriptase inhibitor, 38.9% integrase inhibitor, 2.2% protease inhibitor). The median CD4 cell count was 661 cells/μL (422.5–928 IQR) as shown in the **Table 2.** None of the participants was vaccinated against COVID-19 during the period of sample collection, and 130 samples were positive for anti-SARS-CoV-2 IgG antibody giving a prevalence of 31.0 (26.6–35.7 IC) (**Fig 1)**. The highest seroprevalence of 44.4% was found in December 2021, while the lowest of 0% was recorded in April, September, October and November as shown in **Fig 2.**

**Table 1:** Sociodemographic characteristics of the patients according to the ELISA test result.

Anti-SARS-CoV-2 IgG test result.			
	**Negative, N = 289 (%)**	**Positive, N = 130 (%)**	**Total, N = 419 (%)**
**Sex**			
Female	213.00 (73.70)	97.00 (74.62)	310.00 (73.99)
Male	76.00 (26.30)	33.00 (25.38)	109.00 (26.01)
**Median age (IQR)**	43.00 (35.00, 50.00)	43.00 (35.25, 51.00)	43.00 (35.00, 50.00)
**Residence**			
Rural	34.00 (11.76)	8.00 (6.15)	42.00 (10.02)
Urban	255.00 (88.24)	122.00 (93.85)	377.00 (89.98)
**Occupation**			
Farmers	12.00 (4.15)	4.00 (3.08)	16.00 (3.82)
Retailers	63.00 (21.80)	17.00 (13.08)	80.00 (19.09)
Student	14.00 (4.84)	5.00 (3.85)	19.00 (4.53)
State employees	16.00 (5.54)	9.00 (6.92)	25.00 (5.97)
Housemaids	127.00 (43.94)	71.00 (54.62)	198.00 (47.26)
Others workers*	57.00 (19.72)	24.00 (18.46)	81.00 (19.33)
**Marital status**			
Single	66.00 (22.84)	22.00 (16.92)	88.00 (21.00)
Divorced	25.00 (8.65)	4.00 (3.08)	29.00 (6.92)
Married	149.00 (51.56)	78.00 (60.00)	227.00 (54.18)
Widower	49.00 (16.96)	26.00 (20.00)	75.00 (17.90)
**Education level**			
None	135.00 (46.71)	56.00 (43.08)	191.00 (45.58)
Primary	59.00 (20.42)	31.00 (23.85)	90.00 (21.48)
Secondary	86.00 (29.76)	38.00 (29.23)	124.00 (29.59)
Superior	9.00 (3.11)	5.00 (3.85)	14.00 (3.34)

*Other workers: included bricklayers, plumbers, mechanicians and gardeners

Sociodemographic characteristics categorical variables were expressed as percentages whereas age was summarized with median and interquartile range (IQR); N = number of patients

**Table 2:** Clinical and antiretroviral therapy characteristics of the PLWHIV according to the ELISA test result.

	**Anti-SARS-CoV-2 IgG**		
	**Negative, N = 289 (%)**	**Positive, N = 130 (%)**	**Total, N = 419 (%)**
**Type of HIV**			
**HIV 1**	273.00 (94.46)	121.00 (93.08)	394.00 (94.03)
**HIV 2**	7.00 (2.42)	7.00 (5.38)	14.00 (3.34)
**Coinfection HIV 1&2**	9.00 (3.11)	2.00 (1.54)	11.00 (2.63)
**Time since HIV diagnosis**			
**Median (Q1; Q3)**	11.00 (7.00, 14.00)	12.00 (9.00, 15.00)	11.00 (8.00, 14.00)
**Time on ART**			
**Median (Q1; Q3)**	10.00 (6.00, 14.00)	11.00 (8.00, 14.00)	11.00 (7.00, 14.00)
**Triple regimen strategy**			
**INSTI option**	102.00 (35.29)	61.00 (46.92)	163.00 (38.90)
**NNRTI option**	180.00 (62.28)	67.00 (51.54)	247.00 (58.95)
**PI option**	7.00 (2.42)	2.00 (1.54)	9.00 (2.15)
**CD4 count, cells/mm3**			
**Median (Q1; Q3)**	644.00 (399.00, 921.00)	709.00 (487.00, 948.25)	661.00 (422.50, 928.00)
**Diabetes mellitus**			
**No**	286.00 (98.96)	130.00 (100.00)	416.00 (99.28)
**Yes**	3.00 (1.04)	0.00 (0.00)	3.00 (0.72)
**History of tuberculosis**			
**No**	280.00 (96.89)	127.00 (97.69)	407.00 (97.14)
**Yes**	9.00 (3.11)	3.00 (2.31)	12.00 (2.86)
**Arterial hypertension**			
**No**	280.00 (96.89)	124.00 (95.38)	404.00 (96.42)
**Yes**	9.00 (3.11)	6.00 (4.62)	15.00 (3.58)

Categorical variables were summarized with frequency and percentages whereas continuous variables were summarized with median and Interquartile Range (IQR); N = number of patients

**Abbreviations:** INSTI: Integrase Strand Transfer Inhibitors; PI: Proteinase inhibitor; NNRTI: Non-Nucleoside Reverse Transcriptase Inhibitor, Q1: 1^st^ quartile, Q3: 3^rd^ quartile.

Bivariate analysis indicated that occupation, period of HIV infection diagnosis, type of antiretroviral therapy and CD4 count were significantly (*p*<0.05) associated with SARS-CoV-2 infection (**Tables 3 and 4)**, but, after adjustment for potential confounders, only occupation and type of treatment were remaining as factors associated with SARS-CoV-2 infection. Retailers had half the risk of being infected compared to housemaids with an OR of 0.49 (0.26–0.91; *p* = 0.028). Likewise, the risk of infection was 1.69 times higher in patients on integrase inhibitors compared to that of patients on non-nucleoside reverse transcriptase inhibitors (1.09–2.63; *p* = 0.020) as shown in the **Table 5.**

**Table 3:** Identification of sociodemographic factors associated with SARS-CoV-2 infection in PLWHIV using univariable logistic regression.

	Crude OR	95% CI	*P* value
**Sex**			
Female *	—	—	
Male	0.95	0.59, 1.52	0.844
**Age**	1.01	0.99, 1.02	0.549
**Residence**			
Rural *	—	—	
Urban	2.03	0.96, 4.84	0.082
**Occupation**			
Housemaids*	—	—	
Farmers	0.60	0.16, 1.78	0.386
Retailers	0.48	0.26, 0.87	**0.019**
Students	0.64	0.20, 1.75	0.408
Public sector employees	1.01	0.41, 2.35	0.989
Other workers*	0.75	0.43, 1.31	0.320
**Marital status**			
Single *	—	—	
Divorced	0.48	0.13, 1.41	0.215
Married	1.57	0.91, 2.78	0.111
Widower	1.59	0.81, 3.15	0.179
**Education level**			
None *	—	—	
Primary	1.27	0.74, 2.16	0.386
Secondary	1.07	0.65, 1.74	0.802
Superior	1.34	0.40, 4.06	0.614

*Reference category, Significant at *p*<0.05

*Other workers included: (bricklayers, plumbers, mechanicians, gardeners, and tailors)

Univariable logistic regression model was used to assess factors associated with the risk of a positive serology (IgG anti- SARS-CoV-2) at baseline. Crude odds ratios (OR) and 95% confident intervals (95% CI) were calculated between the dependent variable (SARS-CoV-2 IgG positive/negative) and each of the independent variables (sex, age, residence, occupation, marital status and educational level). Occupation was the only sociodemographic variable significantly associated with SARS-CoV-2 infection.

**Table 4:** Identification of clinical and antiretroviral therapy factors associated with SARS-CoV-2 infection in PLWHIV using univariable logistic regression.

	Crude OR	95% CI	*P* value
**Period of HIV infection**	1.04	1.00, 1.09	**0.047**
**Type of HIV**			
1 *	—	—	
1 and 2	0.50	0.08, 1.98	0.382
2	2.36	0.76, 6.73	0.136
**ART period**	1.02	0.99, 1.05	0.286
**Triple regimen approach**			
NNRTI option *	—	—	
INSTI option	1.61	**1.05, 2.46**	**0.028**
PI option	0.77	0.11, 3.27	0.745
**CD4 count, cells/mm3**	1.00	1.00, 1.00	**0.028**
**Diabetes mellitus**			
No *	—	—	
Yes	0.00		0.978
**History of tuberculosis**			
No *	—	—	
Yes	0.73	0.16, 2.51	0.648
**Arterial hypertension**			
No *	—	—	
Yes	1.51	0.50, 4.27	0.447

*Reference category, Significant at *p*<0.05

**Abbreviations:** ART: Antiretroviral Therapy INSTI: Integrase strand transfer inhibitors; NNRTI: Non-Nucleoside Reverse Transcriptase Inhibitor.

Univariable logistic regression model was used to assess factors associated with the risk of a positive serology (IgG anti- SARS-CoV-2) at baseline. Crude odds ratios (OR) and 95% confident intervals (95% CI) were calculated between the dependent variable (SARS-CoV-2 IgG positive/negative) and each of the independent variables such as arterial hypertension, diabetes and tuberculosis history, anchor drug-based regimen (INSTI-option, NNRTI-option and PI- option), type of HIV (HIV-1, HIV-2, and coinfection HIV-1/2). Period of HIV infection diagnosis, type of antiretroviral therapy and CD4 count were the variables with a *p*-value <0.05 in univariate logistic regression and were then included in the multivariable logistic regression model in **Table 5** (see below) to eliminate the confusional variables.

**Table 5:** Multivariate logistic regression showing the true associated factors with SARS -CoV-2 seropositivity.

Factors associated with SARS -CoV-2 seropositivity	Adjusted OR (aOR)	95% CI	*P* value
**Occupation**			
Housemaids*	—	—	
Farmers	0.74	0.20, 0.27	0.622
Retailers	**0.49**	**0.26, 0.91**	**0.028**
Students	0.67	0.21, 1.86	0.461
Public sector employees	0.94	0.37, 2.23	0.885
Other workers*	0.79	0.44, 1.38	0.410
**Period of HIV infection**	1.02	0.97, 1.06	0.509
**Triple regimen approach**			
NNRTI option *	—	—	
INSTI option	**1.69**	**1.09, 2.63**	**0.020**
PI option	0.80	0.12, 3.50	0.785
**CD4 count, cells/mm3**	1.00	1.00, 1.00	0.056

*Reference category, Significant at *p*<0.05

*Other workers included: (bricklayer, plumber, mechanician, gardener, and tailors)

**Abbreviations:** INSTI: Integrase strand transfer inhibitors; NNRTI: Non-Nucleoside Reverse Transcriptase Inhibitor.

The multivariate logistic regression table (**Table 5**) shows that retailers had half the risk of being infected compared to housemaids (*p* = 0.028, aOR = 0.49, 95% CI = 0.26–0.91). Likewise, the risk of infection was 1.69 times higher in patients on integrase inhibitors compared to that of patients on non-nucleoside reverse transcriptase inhibitors (*p* = 0.020, 95% CI = 1.09–2.63). Adjusted odd ratios (aOR) and 95% CI were calculated after adjustment for confounding factors using multivariate logistic regression.

## Discussion

The findings of this study provided an overview of the state of SARS-CoV-2 seroprevalence in PLWHIV during the first year of COVID-19 pandemic in Bobo-Dioulasso. Although at the outbreak of the pandemic, a worse scenario was predicted for vulnerable populations including PLWHIV [29], but today, in view of the high seroprevalence of 31% (130/419) of antibodies against SARS-CoV-2 in PLWHIV observed in this study, the PLWHIV were not really affected by COVID-19 [30].

As regard seroprevalence of antibodies against SARS-CoV-2 in PLWHIV, our findings show a higher seroprevalence of SARS-CoV-2 in comparison to that reported in November 2 to November 30,^,^ 2020 in Ouagadougou (18%, 36/200), the capital of Burkina Faso [22], in France (13.4%, 254/1901, April 2020 and September 2021) [31], Munich, Germany (1.5%, May and July 2020), Spanish (8.5%, 1^st^ April to 30^th^ September 2020) [21] and in Rome, Italy (0.72%, 8/1106) [32] among PLWHIV approximately in the same period. These low seroprevalences of SARS-CoV-2 infection observed in European countries during the year 2020 could be associated with the compliance and strict observance of confinement instituted early by the European governments compared to African countries. Indeed, at the beginning of the spread of COVID-19 in Burkina Faso, many measures to control the pandemic were taken but some were abandoned due to certain constraints, including financial ones. However, a study conducted in France reported that sub-Saharan African HIV-patients were more likely to have positive SARS-CoV-2-IgG in comparison with PLWHIV originating from France and other countries (OR: 4.78 [95% CI 3.39;6.73], *p*<0.0 0 01) [31], which may reflect social inequalities in health and healthcare in France or the existence of higher levels of pre-existing immunity against SARS-CoV-2 infection for people of sub-Saharan African origin [33, 34].

Considering the SARS-CoV-2 seroprevalence study conducted in the general population in Bobo-Dioulasso, our finding was lower than that reported (55.7%, 95% CI 49·0–62·8) between February and June 2021 [35]. This result could be explained by the existing differences between sampling periods and methods, ART treatments which would favour especially PLWHIV against COVID-19 infection [36], the lower COVID-19 preexisting immunity among people living with HIV [34], and the that PLWHIV could have been vigilant because they perceived themselves to be at higher risk [37]. In contrast, our observation was approximately similar with that of study conducted in Ouagadougou, Burkina Faso, which reported a seroprevalence of 37·4% [95% CI 31·3; 43·5].

The lower seroprevalence recorded in April, September, October, and November among the study participants might be due to the effectiveness of mitigation measures set against the spread of COVID-19. Indeed, one of the measures included the reduction of the PLWHIV who visit the hospital for better biological follow-up. The small amount of plasma samples obtained in April, September, October and November could have explained this low seroprevalence. However, the small amount of blood samples collected in this study was related to the same COVID-19 restrictions, which prevented the PLWHIV to access the healthcare facilities as usual.

Regarding the risk of infection and type of antiretroviral therapy, this study highlighted that PLWHIV on integrase inhibitors had 1.69 times risk to be infected and produce anti-SARS-CoV-2 IgG when compared to those on non-nucleoside reverse transcriptase inhibitors, this is in contrast with the study by Maggiolo et *al*. [38], and the experts of Solidarity trial and the Recovery trial [39] that reported no evidence that any specific antiretroviral therapy regimens affected SARS-CoV-2 infection. However, recent evidence suggests that tenofovir may have antiviral potential against severe COVID-19. [36], but further studies are needed to objectively ascertain the differences in effects of any non-nucleoside reverse transcriptase inhibitors against SARS-CoV-2 infections.

Regarding sociodemographic factors, the housemaids were the social group at high risk of being infected with SARS-CoV-2 in this study. This finding correlates with that of Aden, Yemen [40] and others [41]. Indeed, It was clearly shown that the pandemic affected disproportionately the groups of people who are socially and economically unstable [42]. In contrast, while we found that retailers have low risk to be infected, some authors reported that retailers with direct customer exposure were five times more likely to test positive for SARS-CoV-2 [43]. This study has generated factual data that will contribute in improving response strategies to COVID-19 with keen interest on the risk populations living with HIV.

Nevertheless, our study has some limitations. Small amount of plasma samples was collected from April to November 2021 due to the retrospective method used in this study for collection. This did not enable us to generalized our findings. In addition, to our knowledge, we are the first to use the DS-ЕIA-ANTI-SARS-CoV-2-G (S) kit (Nizhny Novgorod, Russia) for retrospective serosurveillance of SARS-CoV-2 so far in Sub-Saharan Africa. This does not allow us to compare the performance of this test in our context with other studies using the same kit. The collection of additional samples two to three weeks later in subjects negative for anti-SARS-COV-2 IgG could make it possible to exclude ongoing seroconversion by a second ELISA test for confirmation.

## Conclusion

Our study reveals a high seroprevalence of PLWHIV to SARS-CoV- 2 infection during the first year of the pandemic. In addition, these findings provide an additional support of PLWHIV-level immunity against SARS-CoV-2 infection. However, further longitudinal seroepidemiological studies would be relevant to compare the time to peak antibody titer, peak magnitude, and durability of anti–SARS-CoV-2 IgG in people not living with HIV and PLWHIV to investigate whether COVID-19 natural infections may confer comparable antibody immunity in these groups. Finally, although PLWHIV on integrase inhibitors are 1.69 times more likely to be infected than PLWHIV on non-nucleoside inhibitors, this observation remains an intriguing topic that still needs to be clarified.
